# Endovascular Recanalization and Carotid Stenting: The New Approach to Restore Cerebral Perfusion during Aortic Dissection

**DOI:** 10.3390/jcm13092716

**Published:** 2024-05-06

**Authors:** Maxim Agarkov, Kirill Kozlov, Ekaterina Senkina, Sergey Gornov, Natalia Linkova, Elena Kechaeva, Dmitrii Medvedev, Alexander Krasichkov, Anastasiia Dyatlova, Victoria Polyakova

**Affiliations:** 1 Interventional Radiology Gusev Central District Hospital, 56, Moskovskaya Str., Gusev, 238051 Kaliningrad Oblast, Russia; 2 Military Medical Academy of Ministry of Defense of the Russian Federation, 6, Akademica Lebedeva Str., 194044 St. Petersburg, Russia; 3 St. Petersburg Institute of Bioregulation and Gerontology, 3 Dynamo Ave., 197110 St. Petersburg, Russia; 4 Alexander’s Hospital, 4, Solidarnosti Ave., 193312 St. Petersburg, Russia; 5 The Federal Medical-Biological Agency of Russia, 30, Volocolamskoye Highway, 123182 Moscow, Russia; 6 St. Petersburg Research Institute of Phthisiopulmonology, 2-4 Ligovskii Ave., 191036 St. Petersburg, Russia; 7 Department of Radio Engineering Systems of Electrotechnical University LETI, 5F Prof. Popova Str., 197022 St. Petersburg, Russia

**Keywords:** cerebral malperfusion, complication aortic dissection type A, aortic dissection after TAVI, emergency bilateral carotid stenting, retrograde endovascular carotid recanalization

## Abstract

A type A aortic dissection (TAAD) is a dangerous condition requiring emergency surgery. Due to the similarity of the symptoms of cerebral malperfusion in TAAD and the signs of ischemic stroke, a differential diagnosis of these diseases is not always available. Patients with TAAD after cerebral malperfusion can have a neurological deficit. Thrombolysis is performed in this case. It can worsen the patient’s condition and increase the risk of mortality and disability. The aim of the study is to evaluate the new approach to restoring cerebral perfusion during aortic dissection. This approach includes endovascular recanalization and carotid stenting. Methods: Two clinical cases of TAAD complicated by cerebral malperfusion are described. The first patient is 73 years old and was admitted as planned to perform transcatheter aortic valve implantation (TAVI) for grade III aortic stenosis. The patient underwent transcatheter aortic valve implantation (TAVI) on the second day after admission. The second patient is 60 years old and was hospitalized by an ambulance with strong hypertension and ischemia. The surgical correction of aortic dissection was postponed until the neurological status assessment in both patients. Results: The surgery to correct the aorta dissection was deemed inappropriate. The carotid arteries have been reanalyzed, and cerebral perfusion has been restored in a short time in both patients. Conclusion: Acute bilateral internal carotid occlusion is a potentially fatal TAAD outcome. Emergency endovascular recanalization and carotid stenting may be considered one of the few ways to restore cerebral perfusion.

## 1. Introduction

Type A aortic dissection is a common disease that can lead to dangerous consequences and requires emergency surgical interventions [1,2]. The complexity of pathology is aggravated by frequent complications, such as hemopericardium with tamponade, acute coronary occlusion, malperfusion syndrome, and acute aortic valve insufficiency. Eleven percent of TAAD cases are complicated by cerebral malperfusion (CM) [3,4]. According to published data, the presence of CM in perioperative period increases hospital mortality from 9.5 to 50%, and the risk of perioperative stroke from 4.5 to 46.7%, the occurrence of coma from 4.5 to 40% and decreased consciousness from 29.6 to 73.3% [5]. In case of CM the primary goal of surgical treatment is an adequate perfusion restoration to avoid irreversible brain damage.

The risk of coma increases from 4.5 to 40%, and decreased consciousness from 29.6 to 73.3% [5]. TAAD in patients with acute CM is variable and depends on the degree of neurological deficit [6]. Symptoms can range from a mild neurological deficit to coma and initially can be interpreted as an ischemic stroke. Clinical guidelines for patients with ischemic stroke allow thrombolytic therapy (TLT) with no angiography if the total time from symptom onset is less than 4.5 h [7]. Multispiral CT (MSCT) before hospitalization performed in a mobile computed tomography complex does not allow the visualization of the whole aorta with its root due to the low aperture. Thus, in patients with CM and neurological deficits caused by a TAAD, the TLT could be developed, which would dramatically exacerbate the prognosis.

In addition, in the presence of aortic arch fenestration and/or dissection spread to the branches of the aortic arch, the symptoms of CM can develop not only at the time of the dissection but also in the postoperative period. It has been shown that carotid dissection persists in 15–41% of cases after the surgical treatment of acute aortic dissection [8].

Unlike the infra-renal lesion, where endovascular techniques are commonly used, the treatment of ascending aorta dissection is available only as a part of emergency cardiac surgery. The classical approach consists of aortic arch prosthetics when the blood flow is restored through the true carotid lumen. However, surgical interventions in cases of aortic and brachial pathology combinations are accompanied by extremely high mortality and neurological complications. Less radical surgical interventions, such as supracoronary ascending aorta prosthetics or the David Bental de Bono procedure, show satisfactory results, especially in acute forms of the disease, but the prognosis for patients with persistent aortic arch and/or main artery dissection remains doubtful [9].

The development of surgical and perfusion techniques made possible a new approach to combined interventions. Some reports describe the carotid stenting (CS) as a first stage before the surgical aorta prosthetics [10,11,12,13]. The purpose of CS is to restore blood flow and reveal acute cerebral ischemia. This approach minimizes the time of cerebral ischemia and increases the chances of a good long-term prognosis.

Cerebral ischemia could be a result of the delay in the established diagnosis of TAAD [14]. Then, an ischemic stroke accompanying aortic dissection may reduce awareness of the stroke unit. The low level of consciousness and speech and language alterations may hinder or prevent the detection of acute chest pain. In addition, chest X-rays showed mediastinal widening or aortic enlargement in only 50% of cases. This explains the difficulty of detecting aortic dissections manifesting themselves in neurological symptoms and the consequent higher mortality rates (30%) compared to those seen in other cases of aortic dissection (22.6%). Missed diagnosis may also lead to thrombolysis, which is recommended now in an ischemic stroke, with a mortality rate of 71% in patients receiving rTPA [15].

In this article, we are describing two clinical cases from two different medical centers that were performed by one surgeon. In four years, more than 500 thrombectomy procedures were performed in the mentioned two centers in patients with stroke. In two cases (0.4%), the CS was performed to restore blood flow through the true artery lumen, which was occluded by a hematoma of the false lumen during the TAAD.

**Clinical Case 1**

**Figure 1:**
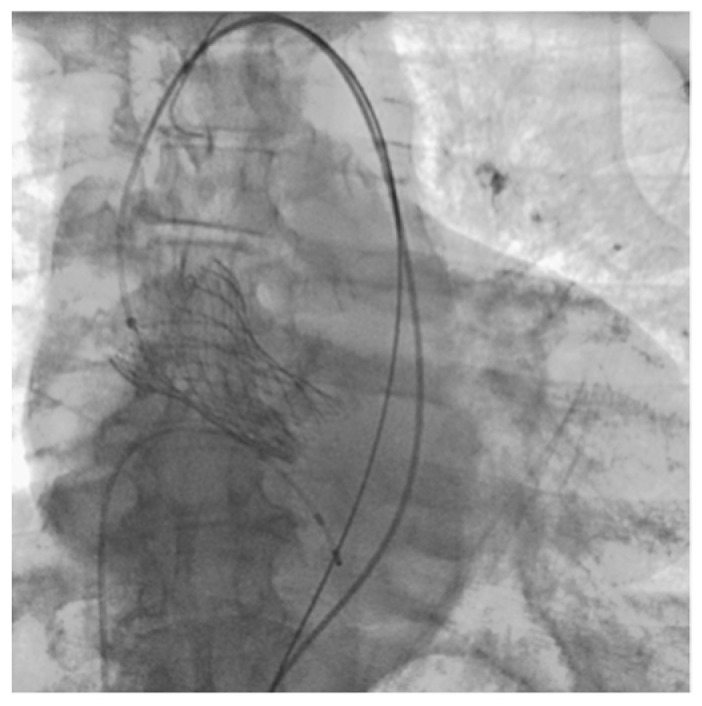
Final shooting after TAVI (Clinical Case 1, Patient M., 73 years old).

Patient M., a male, 73 years old, was admitted as planned to perform transcatheter aortic valve implantation (TAVI) for grade III aortic stenosis (mean gradient 41 mmHg, Aortic Valve Area index 0.44 cm^2^/m^2^). The patient has been suffering from 4th stage CKD for a long time, and the condition after surgical treatment and chemoradiotherapy for laryngeal cancer. The patient underwent the TAVI on the second day after hospitalization. The operation was performed under regional anesthesia and was performed normally (Figure 1).

In the first case, thrombolytic therapy was not considered, since the patient underwent a transcatheter aortic valve replacement procedure three weeks before the episode. The refusal to perform computed angiography after performing native computed tomography when the symptoms of an acute cerebrovascular accident appeared was a diagnostic flaw of the medical team. The operating surgeon regarded the symptoms as the acute cerebrovascular accident associated with embolism, vegetation from an inflamed heart valve. In order to reduce the time for thrombectomy, it was decided to abandon computed angiography. The surgeon also refused computer angiography due to the fact that he owned the data of a previously performed computer angiography. This study was performed before TAVI. The TAVI procedure and carotid artery stenting were performed by one surgeon.

**Figure 2:**
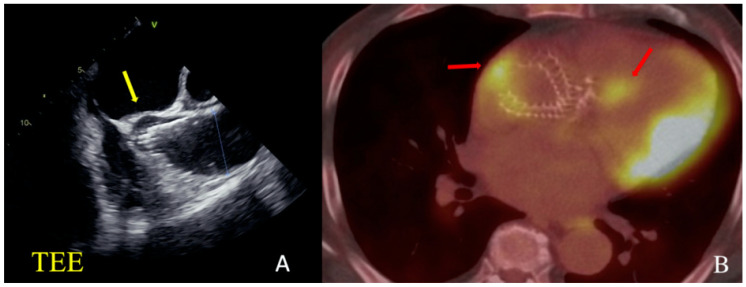
Clinical Case 1, Patient M., 73 years old. (**A**) TE ECHO visualization. The yellow arrow indicates a suspected abscess of non-coronary sinus. (**B**) PET results. The red arrow indicates the zones of hypermetabolism in the area of the valve crown and coronary sinus.

However, on the 10th day after TAVI, the patient noted a deterioration of statement, experiencing a fever of up to 39 °C. According to transesophageal echocardiography (TE-ECHO), a thrombus/vegetation of 6 mm was visualized on the previously implanted valve leaflets. A PET-CT revealed a focus of inflammation on the valve prosthesis and in the area of the non-coronary sinus (Figure 2).

**Figure 3:**
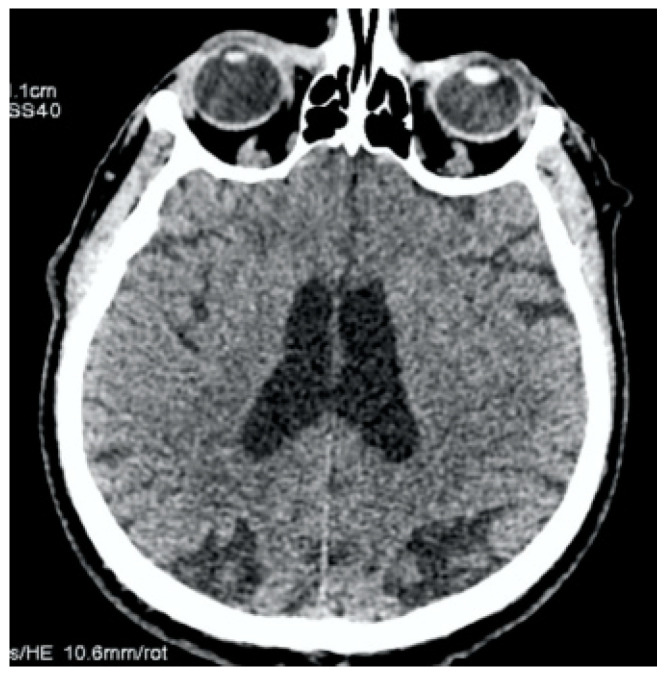
Clinical Case 1, Patient M., 73 years old. Brain MSCT in 20 min after symptoms’ onset. No data for ischemia or brain damage; ASPECTS = 10.

The antibiotic therapy was prescribed by the next scheme: Vancomycin 1 g taken 2 times per day and Gentamicin 80 mg taken 3 times per day, as well as symptomatic treatment. In the next two weeks, the patient noted an improvement and the temperature normalized. However, on the day of discharge, in the presence of medical personnel, the patient developed generalized convulsive syndrome, stopped by intravenous diazepam administration and complicated by left-sided hemiplegia. Immediately, the MSCT was performed. There were no signs of stroke, ASPECTS = 10 (Figure 3).

**Figure 4:**
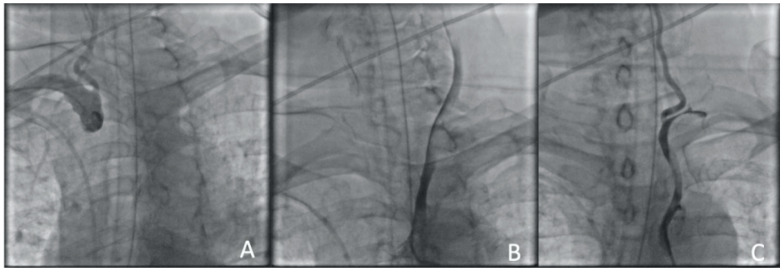
Clinical Case 1, Patient M., 73 years old. Results of the angiography: (**A**) brachiocephalic and subclavian artery angiography via radial access; (**B**) left common carotid artery angiography, a low contrast defect is visible throughout; (**C**) left subclavian angiography. A contrast defect is visible throughout the artery.

According to the presence of thrombus or vegetation on the implanted valve, a right medial cerebral artery thrombotic occlusion was suspected. The patient was immediately placed in the operating room. The total time from the moment of the symptoms’ onset to arterial access was less than 30 min. Using cerebral angiography, it has been revealed that there is a type A aortic dissection (Stanford), with both common carotid arteries occlusions (Figure 4).

**Figure 5:**
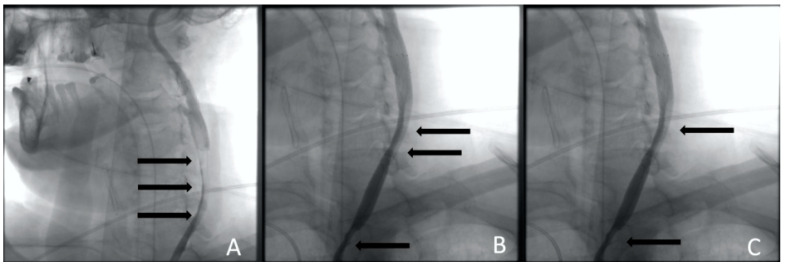
Clinical Case 1, Patient M., 73 years old. Results of the angiography: (**A**) left ICA stenting with the first stent; (**B**) CCA stenting with a second stent; (**C**) CCA stenting with the third stent. The arrows indicate contrast defects that persist after stenting (compression of the true lumen).

After the emergency examination by the cardiac surgeon and neurologist, a decision of carotid revascularization and stenting was performed. Through the right common femoral artery, with no technical difficulties, the revascularization of the left common carotid artery (CCA) by the implantation of three self-expending carotid stents into the left internal carotid artery (ICA) and CCA was performed (Figure 5).

**Figure 6:**
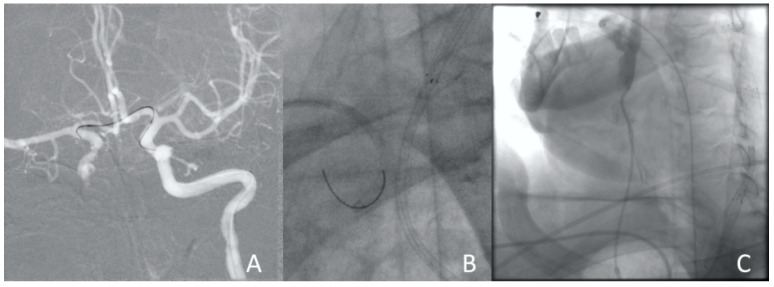
Clinical Case 1, Patient M., 73 years old. Results of the angiography: (**A**) conducting the guide catheter through the ACA and further into the ICA; (**B**) the moment of capturing the guide catheter from the radial artery; (**C**) right ICA angiography using the diagnostic catheter via radial access.

The left hemisphere blood flow was restored at the level of TICI-III. However, repeated attempts to cannulate the right CCA were unsuccessful due to orifice compression. It was decided to perform the retrograde recanalization of the right ICA and CCA through the anterior connective artery (ACA). Subsequently, the guide catheter of 0.014 Fr was (doubtful) withdrawn into the aorta, captured using a loop trap, and externalized via radial access (Figure 6).

**Figure 7:**
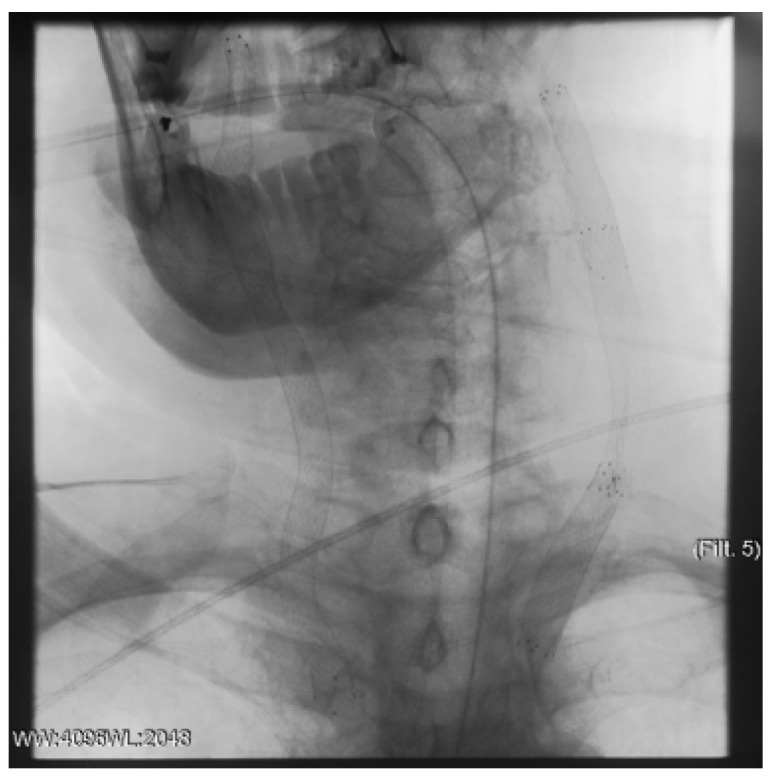
Clinical Case 1, Patient M., man, 73 years old. Full metal jacket of both sides’ carotid arteries.

The right ICA and CCA were also stented using the peripheral self-expending stent 6.0 × 150 Innova Boston Scientific via radial access. Cerebral circulation was totally restored. The time from symptom onset to left and right hemisphere revascularization was 55 and 150 min, respectively (Figure 7).

After the operation, the MSCT with angiographic control was performed. It confirmed the TAAD. Cerebral circulation was preserved. The surgical correction of the aortic dissection was postponed until the neurological status assessment. In 24 h after reperfusion, the repeated MSCT was performed.

The patient underwent an MSCT scan of the brain without contrast 30 h later. MSCT was performed against the background of a progressively deteriorating patient’s condition with the development of multiple organ failure syndrome. According to MSCT, signs of bilateral edema and ischemic brain damage were observed: one point in the left hemisphere and zero points in the right hemisphere on the ASPECTS scale. In the future, no additional studies were performed on the patient. The data presented earlier were taken from the archive of the Department of Interventional Radiology. The data of the latest MSCT were not provided due to the expiration of 3 years and their automatic deletion from the database according to the protocol of the hospital, developed in accordance with the legal norms of archiving digital images of the medical documentation of the Russian Federation.

**Figure 8:**
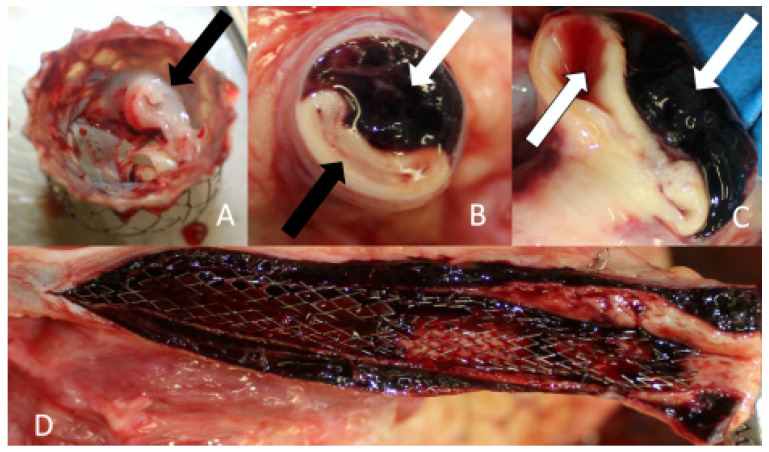
Clinical Case 1, Patient M., 73 years old. Results of the autopsy: (**A**) aortic valve prosthesis with vegetation (black arrow); (**B**) left CCA. The compression of true lumen (black arrow) by subintimal hematoma (white arrow); (**C**) brachiocephalic trunk. The compression of true lumen (black arrow) by subintimal hematoma (white arrow); (**D**) left CCA. The dissemination of hematoma to the external and internal carotid arteries.

Considering the progressive brain swelling and signs of severe brain damage, the surgery to correct the aorta dissection was deemed inappropriate. The patient passed away 72 h after the onset of symptoms. The cause of death of the first patient was progressive cerebral edema. At the autopsy, the compression of stents by a false lumen hematoma was revealed (Figure 8).

**Figure 9:**
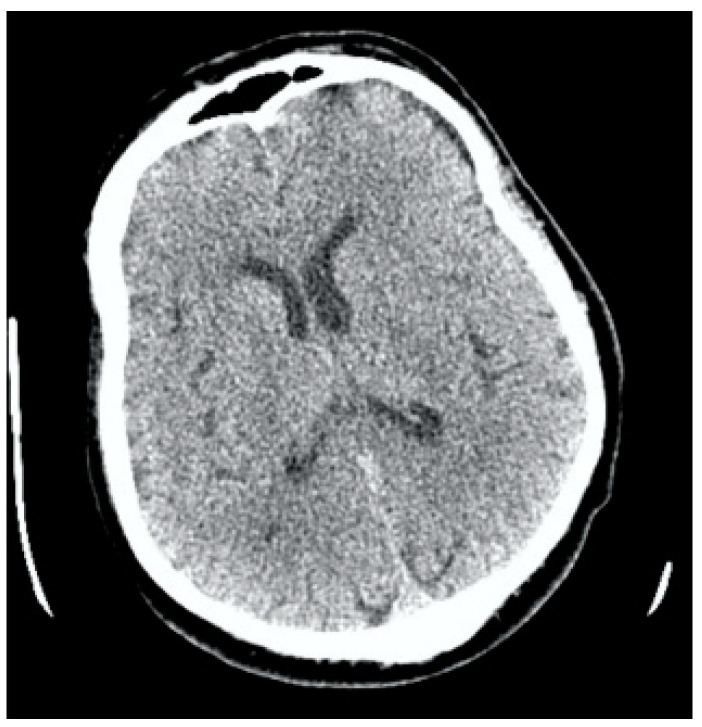
Clinical Case 2, Patient S., male, 60 years old. Cerebral MSCT in 30 min after the onset of symptoms. There are no data for ischemia and brain damage, ASPECTS = 10.

**Clinical Case 2.** Patient S., a 60-year-old male, was hospitalized by ambulance from a public place (pharmacy). At the time of admission, the blood pressure was 210/130 mm Hg. The patient had right-sided hemiplegia, smoothness of the nasolabial fold, motor aphasia, and showed 14 points on the NIH scale. The hypertension correction was made by the intravenous administration of Urapidil at a dosage of 10 mg. The patient got an emergency MSCT, which showed the absence of ischemic changes on the ASPECTS scale of 10 points (Figure 9).

**Figure 10:**
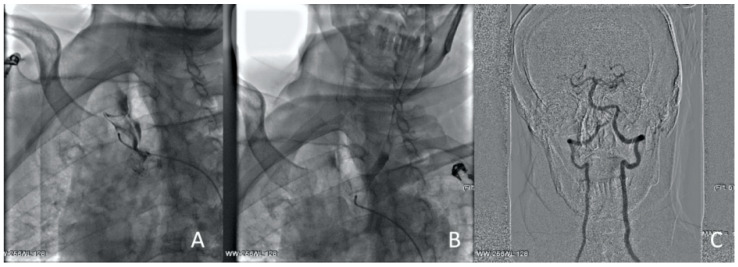
Clinical Case 2, Patient S., male, 60 years old. Aortic arch angiography. (**A**) BCT dissection with right CCA and right subclavian artery occlusion; (**B**) left CCA angiography determines the true lumen compression and a stop of blood flow through the ICA; (**C**) left subclavian artery angiography determines the stealphenomenon throughout the vertebral arteries.

TLT was started immediately. Considering the progressive increase in neurological deficit within 10 min from the start of TLT, it was decided to perform a combined reperfusion. The patient was taken to the cath lab 60 min after the onset of the symptoms. The first difficulty was performing a puncture of the right femoral artery. Despite the presence of a clear pulsation and the pulsating blood flow from the needle, the guidewire could not be placed in the artery lumen. To perform the immediate reperfusion, it was decided to change access on the left side, which was performed without technical difficulties. Angiography showed a total aortic dissection from the bifurcation to the right iliac artery (Figure 10). Selective cerebral angiography showed brachiocephalic trunk (BCT) occlusion, left CCA occlusion, and dynamic left subclavian artery compression. The patient also demonstrated a stealphenomenon along the vertebral arteries from left to right (Figure 10).

**Figure 11:**
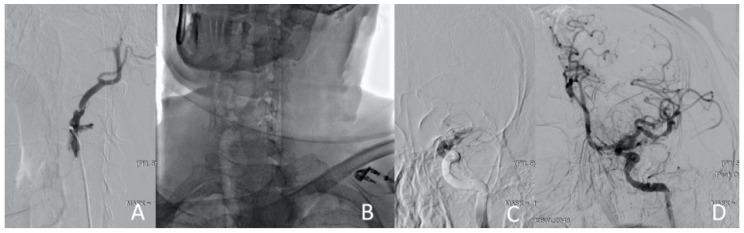
Clinical Case 2, Patient S., male, 60 years old. Results of the angiography: (**A**) left ICA occlusion from the bifurcation, the external carotid artery is passable; (**B**) left ICA stenting with the stent protrusion into the aorta; (**C**) stagnation of contrast in ICA due to a thrombus in the C1 segment of the ICA; (**D**) restoration of blood flow through the cerebral arteries.

Considering the surgeon’s experience working with such patients (Clinical Case 1) as well as endovascular team engagement, it was decided to perform emergency endovascular cerebral reperfusion. The patient, without technical difficulties, got a self-expanding stent measuring 6.0 × 150 mm in the left internal and common carotid arteries with protrusion into the aorta; however, control angiography revealed the thrombotic occlusion of the ICA in segment C7. The thromboaspiration using a catheter (6Fr) was performed. Blood flow was restored at the level of TI-CI 2b-3 (Figure 11).

**Figure 12:**
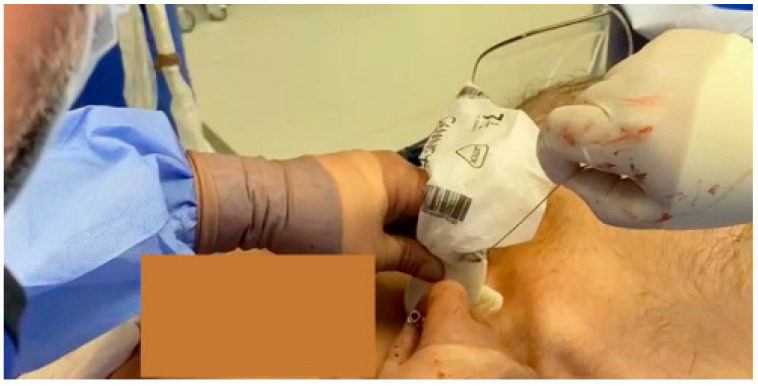
Clinical Case 2, Patient S., male, 60 years old. Retrograde puncture of the right ICA.

The left hemisphere circulation was restored in 90 min from the onset of the symptoms. The antegrade cannulation of the right CCA was technically impossible due to true lumen compression. Within 5 min, a retrograde puncture of the right CCA under ultrasound control was performed (Figure 12).

The guide catheter was introduced into the descending aorta, and then into the left iliac artery, where it was externalized. It was decided not to remove the retrograde guide catheter from the CCA before installing the stent, since the compression during hemostasis could complicate the procedure. On the second guide catheter from the ICA orifice with protrusion into the aorta, a self-opening stent of 6.0 × 120 mm was implanted.

**Figure 13:**
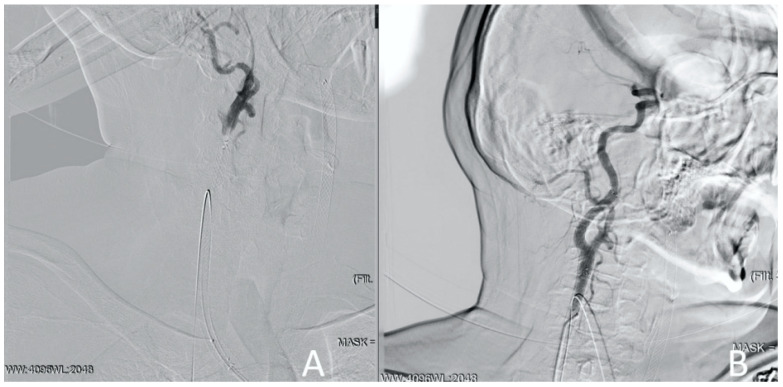
Clinical Case 2, Patient S., male, 60 years old. Right CCA revascularization. (**A**) Implantation of a 6.0 × 120 mm stent in CCA. No blood flow in ICA due to hematoma transition to the C1 segment. (**B**) Cone stent 8.0/10.0 × 40 mm implantation. Right hemisphere blood flow to TICI 3 level was totally restored.

However, control angiography showed the absence of blood flow through the ICA due to the spread of hematoma to the C1 segment of the ICA. A cone stent of 8.0/10.0 × 40 mm was positioned in the ICA, with previously implanted stent overlap. The blood flow was totally restored. Right hemisphere revascularization was performed at 140 min from the onset of symptoms. Control angiography showed a complete restoration of cerebral circulation to the level of TICI-III (Figure 13).

The guide catheter from the right ICA was removed. Hemostasis was performed by manual pressing.

**Figure 14:**
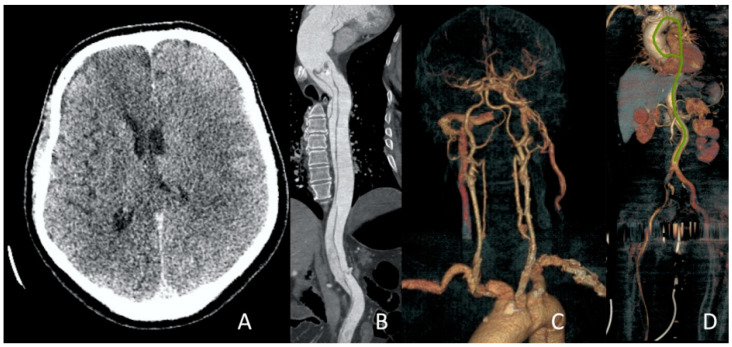
Clinical Case 2, Patient S., male, 60 years old. MSCT angiography in 24 h after revascularization. (**A**) Brain edema of both hemispheres was determined; (**B**) MSCT aortography showed the type A aortic dissection; (**C**) circulation through the cerebral arteries is preserved; (**D**) circulation in the right lower limb is preserved.

The visual assessment of the right lower limb showed the pallor of the skin. According to an ultrasound examination, the blood flow through the superficial femoral artery was slowed down. The decision on surgical aorta prosthetics was also postponed until the assessment of brain damage. The neurological deficit according to the NIH scale at the end of revascularization remained at 29 points; according to the Glasgow Coma Scale, it was 7 points. A repeated CT performed in 24 h showed severe cerebral edema and ischemic brain damage. ASPECT = 2 (Figure 14).

The angiography showed stents patency and cerebral circulation presence. The patient passed on the 3rd day because of progressive cerebral edema. The autopsy was not performed for religious reasons.

## 2. Discussion

Acute CM is an unfavorable prognostic sign in patients with a TAAD and, according to the literature data, reaches 100% mortality in cases of conservative management [16,17]. For a long time, surgical interventions in the aorta were limited by the presence of severe neurological deficits. A few reports showed low survival in patients hospitalized in coma [6,18,19]. However, several recent reports have shown that endovascular reperfusion as a first stage can improve the prognosis, especially if the time from symptom onset is less than 5 h [20,21,22].

We have not found a literature description of bilateral interventions in cases of cerebral occlusion in patients with a TAAD. However, several case reports have been published after a successful technical revascularization of bilateral ICA thrombotic occlusion, which demonstrate both positive and unfavorable outcomes [23,24,25,26]. Studies suggest that the results of mechanical thrombectomy and stenting may depend on the patient’s clinical condition, collateral circulation, and localization of occlusion [26,27,28,29]. However, one of the main factors influencing the CM outcome due to the TAAD is the time that was taken for reperfusion [30]. A main feature of favorable outcomes was the short time from symptom onset to revascularization [31].

The condition of both patients was urgently discussed with vascular surgeons, given that both patients had severe neurological symptoms. The first patient was in a coma, showing seven points on the Glasgow scale. The second patient had hemiplegia and then entered a coma, showing 9 points on the Glasgow scale. It was decided to initially restore blood flow through the carotid arteries using stenting, followed by neurological status monitoring. In case of the partial or complete regression of neurological symptoms, it was planned to perform aortic prosthetics. The choice of tactics for the endovascular revascularization of two carotid arteries was presumably due to a faster recovery time of blood flow through two carotid arteries in comparison with open aortic prosthetics.

In both clinical cases described above, there were significant difficulties with the right CCA catheterization. However, the technique of retrograde guide catheter conduction with externalization seems to be a good and fast enough alternative.

Another important point is choosing a stent type. A pathoanatomic examination revealed stent compression by false lumens. That demonstrates the insufficient radial force needed to maintain the patency of the true lumen. In case of signs of compression, it is better to use balloon-expandable stents. So, Mukherjee et al. described three successful clinical cases, where retrograde carotid arteries stenting was performed after the aortic plasticity with residual dissection BCA stenosis. Venous stents (Boston Scientific VICI) were chosen because of their greater radial strength in comparison to commonly used stents, with the aim of better false lumen obliteration [32].

In addition, the need for double antiplatelet therapy in the postoperative period may lead to a postponement of emergency aortic surgery, which can only be performed with a single antiaggregant agent [13].

## 3. Conclusions

Acute bilateral internal carotid arteries occlusion is a potentially fatal complication of ascending aorta dissection. Understanding a very poor prognosis necessitates the use of any possible techniques to increase the chances of patient survival. An emergency endovascular procedure can be considered one of the few ways to restore cerebral circulation.

## Data Availability

The datasets presented in this article are not available because it contains the confidential information about patients.
